# Editorial: Neurocomputational models of decision-making and cognitive processes

**DOI:** 10.3389/fnhum.2026.1775871

**Published:** 2026-01-23

**Authors:** Lidia Ghosh, Anuradha Saha

**Affiliations:** 1 Department of Computer Application, RCC Institute of Information Technology, Kolkata, India; 2 Department of Applied Electronics & Instrumentation Engineering, Netaji Subhas Engineering College, Kolkata, India

**Keywords:** Balloon Analogue Risk Task (BART), EEG, functional Near-Infrared Spectroscopy (fNIRS), neurocomputation, Weber's Law

## Introduction

Understanding how cognitive processes emerge from neural computations remains a central challenge in neuroscience. Computational modeling offers a rigorous framework for integrating neurophysiological, behavioral, and cognitive data to elucidate mechanisms underlying perception, decision-making, and adaptive behavior. The Research Topic “*Neurocomputational Models of Decision-Making and Cognitive Processes*” brings together interdisciplinary contributions that employ neurocomputational approaches to investigate the neural and cognitive bases of recognition, reward processing, risk evaluation, and interoceptive decision-making. Collectively, the five accepted articles highlight innovative computational methods, from recurrent neural networks to homeostatic reinforcement learning models, and demonstrate their utility in explaining complex cognitive and behavioral phenomena.

## How short video addiction affects risk decision-making behavior in college students based on fNIRS technology

In Zhang et al., the authors investigate how short video addiction influences risk-based decision-making in college students using functional Near-Infrared Spectroscopy (fNIRS) during the Balloon Analogue Risk Task (BART). Behavioral results indicate that individuals with short video addiction display heightened risk-taking tendencies, shorter reaction times, and increased balloon explosions, particularly under short video cue conditions. Neurophysiological data reveal enhanced activation in the right orbitofrontal cortex (OFC) and right frontopolar area (FPA) during risky decisions with cue exposure, while the control group shows no such activations. Furthermore, individuals with addiction exhibited pronounced sensitivity to loss outcomes, engaging the left dorsolateral prefrontal cortex (DLPFC) and FPA. The findings illustrate how environmental cues modulate risk decision-making and highlight the utility of combining computational and neuroimaging approaches to understand the neural mechanisms underlying behavioral addictions.

## Weber's Law as the emergent phenomenon of choices based on global inhibition

In Penconek, the emergence of Weber's Law is examined through a computational lens. Using a recurrent attractor network with binary neurons, the study tests the hypothesis that Weber-like proportional discrimination arises from choice circuits employing global inhibition. The analysis shows that Weber's Law holds approximately for near-threshold discriminations but is violated for high-probability, easy discriminations, replicating the convex relationship between stimulus intensity and Weber fractions observed empirically. While the model captures the general pattern of deviations, it does not fully account for low-intensity violations, suggesting that additional neuronal mechanisms, such as speed–accuracy trade-offs, may contribute. This work exemplifies how neurocomputational modeling can formalize classic psychophysical laws and generate mechanistic explanations for empirical patterns.

## Forgetting phenomena in the Iowa Gambling Task: a new computational model among diverse participants

Yang et al. introduces the Exploitation and Exploration with Forgetting (EEF) model to capture dynamic decision-making in the Iowa Gambling Task (IGT). The model integrates a forgetting parameter into decision processes, alongside participants' initial deck preferences, allowing it to account for information decay and its influence on exploitation-exploration trade-offs. Analyses of 504 participants demonstrate that the EEF model provides superior fit compared to prior models and captures behavioral dynamics across diverse participant groups. Novel metrics, Sequential Exploration Decay (SED) and Forgetting Interval (FI), quantify the role of memory decay in decision strategies. Extensions of the model highlight how age and gambling frequency modulate forgetting effects, providing insights into cognitive changes across the lifespan. This contribution underscores the importance of incorporating temporal dynamics of memory and forgetting into computational frameworks for decision-making.

## Heterogeneous appetite patterns in depression: computational modeling of nutritional interoception, reward processing, and decision-making

In Uchida et al., the authors apply homeostatic reinforcement learning (HRL) models to investigate appetite dysregulation in depression. The study formalizes the relationship between internal interoceptive states, reward processing, and behavior. Simulation experiments reveal that reduced interoception decreases reward system activity and increases punishment, mirroring decreased appetite observed in certain depressive subtypes. Conversely, enhanced interoception increases reward activity but impairs goal-directed behavior, reflecting increased appetite. Comparisons with standard reinforcement learning parameters, such as inverse temperature (β) and delay discount factor (γ), suggest that these parameters modulate homeostatic regulation in ways similar to interoceptive alterations. This work exemplifies the application of computational psychiatry to link neurophysiology, decision-making, and clinical phenotypes, offering insights for targeted interventions and subtype classification in depression.

## Conclusion

The Research Topic “*Neurocomputational Models of Decision-Making and Cognitive Processes*” exemplifies the growing convergence of computational modeling, cognitive neuroscience, and behavioral research. By providing mechanistic accounts of recognition, risk evaluation, memory decay, and interoceptive regulation, the contributions collectively illuminate the neural and computational bases of decision-making and cognitive control. The Research Topic underscores the potential of neurocomputational approaches to inform theory, guide experimental design, and ultimately support translational applications in cognitive and clinical neuroscience.

